# Food Allergy and Intolerance: A Narrative Review on Nutritional Concerns

**DOI:** 10.3390/nu13051638

**Published:** 2021-05-13

**Authors:** Domenico Gargano, Ramapraba Appanna, Antonella Santonicola, Fabio De Bartolomeis, Cristiana Stellato, Antonella Cianferoni, Vincenzo Casolaro, Paola Iovino

**Affiliations:** 1 Allergy and Clinical Immunology Unit, San Giuseppe Moscati Hospital, 83100 Avellino, Italy; dogargano4920@aosgmoscati.av.it (D.G.); fadebartolomeis5187@aosgmoscati.av.it (F.D.B.); 2 Department of Medicine, Surgery and Dentistry “Scuola Medica Salernitana”, University of Salerno, 84081 Baronissi, Italy; rappanna@unisa.it (R.A.); asantonicola@unisa.it (A.S.); cstellato@unisa.it (C.S.); vcasolaro@unisa.it (V.C.); 3 Division of Allergy and Immunology, The Children’s Hospital of Philadelphia, Perelman School of Medicine at University of Pennsylvania, Philadelphia, PA 19104, USA; cianferonia@chop.edu

**Keywords:** food allergy, food intolerance, nutrition, nutritional concerns

## Abstract

Adverse food reactions include immune-mediated food allergies and non-immune-mediated intolerances. However, this distinction and the involvement of different pathogenetic mechanisms are often confused. Furthermore, there is a discrepancy between the perceived vs. actual prevalence of immune-mediated food allergies and non-immune reactions to food that are extremely common. The risk of an inappropriate approach to their correct identification can lead to inappropriate diets with severe nutritional deficiencies. This narrative review provides an outline of the pathophysiologic and clinical features of immune and non-immune adverse reactions to food—along with general diagnostic and therapeutic strategies. Special emphasis is placed on specific nutritional concerns for each of these conditions from the combined point of view of gastroenterology and immunology, in an attempt to offer a useful tool to practicing physicians in discriminating these diverging disease entities and planning their correct management. We conclude that a correct diagnostic approach and dietary control of both immune- and non-immune-mediated food-induced diseases might minimize the nutritional gaps in these patients, thus helping to improve their quality of life and reduce the economic costs of their management.

## 1. Introduction

**Figure 1:**
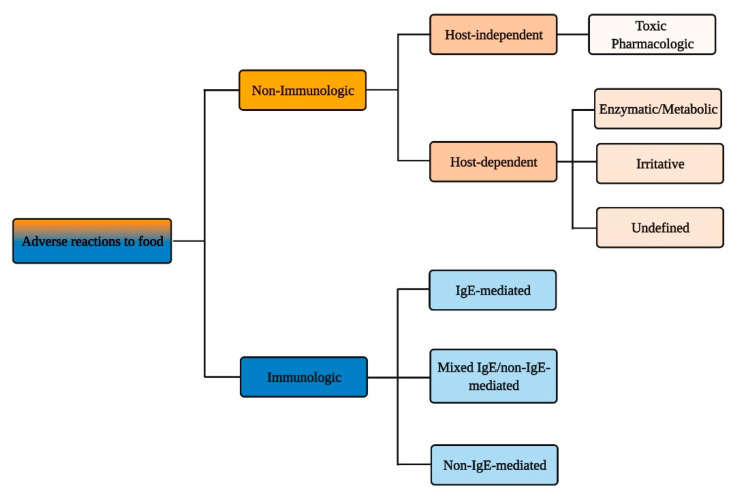
Immunologic vs. non-immunologic adverse reactions to food.

According to the authoritative definition issued in 2010 by an Expert Panel Report sponsored by the National Institute of Allergy and Infectious Diseases (NIAID), food allergy is defined as “an adverse health effect arising from a specific immune response that occurs reproducibly on exposure to a given food” and food intolerance as “nonimmune reactions that include metabolic, toxic, pharmacologic, and undefined mechanisms” [1]. Yet, this distinction and the diversity of events and pathologies that lies behind it (Figure 1) is not clearly embodied in the public perception of the adverse reactions that can occur following food intake.

It is well established that the prevalence of true IgE-mediated food allergy is significantly less common than food allergy identified as self-reported disease, yet even epidemiological data reflect the difficulty in the identification of bona fide IgE-mediated allergy, as several reports do not include a clinical confirmation of disease [2].

Stringent surveys of food allergy prevalence indicate, at least in westernized countries, a trend towards greater persistence of pediatric food allergies and higher rates of adult-onset cases than previously appreciated [2,3]. The impact of therapeutic food allergy regimens on nutritional needs will therefore need to be adjusted according to this expanding spectrum [4], as this is the cornerstone of all types of food allergy prevention, from primary to tertiary [5]. This evolving landscape for immediate hypersensitivity reactions to food, as well as increased knowledge on non-IgE- or mixed IgE/non-IgE immunological responses brings increasing nutritional challenges for diseases in which long-term food elimination is a primary therapeutic strategy [6], underscoring the key role of a correct dietary approach strictly driven by appropriate diagnostics [7].

In this narrative review, the pathophysiological and clinical features of immune and non-immune adverse reactions to food—along with general diagnostic and therapeutic strategies—are outlined; in particular, the specific nutritional concerns are discussed from the combined point of view of gastroenterology and immunology, in an attempt to offer a useful tool to practicing physicians in discriminating these very different disease entities and in planning their correct management.

## 2. Immunologic Adverse Reactions to Food

**Figure 2:**
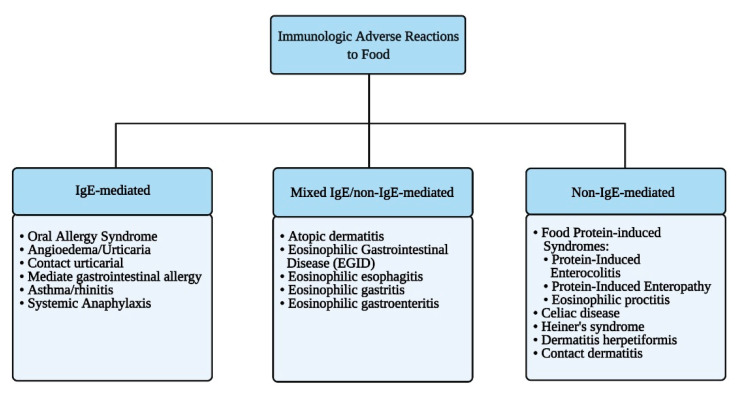
Immunologic adverse reactions to food.

Immune-mediated reactions to food can be classified depending on the involvement of IgE-mediated and/or other immune responses to ingested antigens (Figure 2).

### 2.1. IgE-Mediated Food Allergy

IgE-mediated food allergy is generally characterized by its rapid onset, which can be within minutes to an hour after ingesting the allergen, according to the World Allergy Organization (WAO). The signs and symptoms can be mild and localized to mucocutaneous manifestations or can involve different systems that are concomitantly affected in systemic anaphylaxis (Table 1).

The gastrointestinal (GI) tract is a common target for immediate hypersensitivity reactions to food [1,11,12] with the main clinical presentations being the oral allergic syndrome (OAS) and the symptoms of immediate GI hypersensitivity. OAS, also known as pollen-food syndrome, is characterized by itching and tingling sensation of the oral mucosa and/or upper pharynx, the erythema of the perioral and oral mucosa with mild edema that occurs within minutes from ingestion of some foods, especially fresh fruits, and vegetables [13]. This localized reaction occurs primarily in patients with respiratory allergies (rhinoconjunctivitis, asthma) that have specific IgE directed against panallergens, which are proteins featuring homologous epitopes present in seasonal or perennial aeroallergens (such as pollens) and certain foods—mostly fruits and vegetables [14,15]. Patients with birch-pollen hay fever may have OAS symptoms after ingesting hazelnut, apple, carrot, and celery, whereas patients with IgE-mediated sensitivity to ragweed pollen may react to melons (e.g., watermelon or cantaloupe) and banana. The signs and symptoms rarely involve areas beyond those directly involved in the first contact with the culprit food and are mostly self-limiting. This occurs as the epitopes involved are conformational and thus are recognized by specific IgE only in the native form of the protein and no longer once denatured by gastric low pH. Accordingly, most patients with OAS can tolerate the triggering food when it is consumed cooked, as epitopes are destroyed by the heating process. These are important features differentiating OAS from an initial presentation of a more generalized allergic reaction, such as an urticaria/angioedema developing either as an isolated cutaneous reaction or as part of an anaphylactic response.

The symptoms of immediate GI hypersensitivity include nausea, abdominal pain, cramps, vomiting, and/or watery/mucous diarrhea. Acute vomiting is the most common presentation and the one best documented as immunological and IgE-mediated. Other IgE-mediated food-induced allergic manifestations are listed in Table 1.

Anaphylaxis is a serious allergic reaction that is rapid in onset and may be life-threatening [16]. It can be mediated by IgE, or by other immunological or non-immunological mechanisms [17]. According to the current NIAID criteria, anaphylaxis is highly likely when there is the acute onset of an illness (minutes to several hours) with either (1) cutaneous manifestations associated with the involvement of respiratory or cardiovascular system (dizziness, weakness, tachycardia, hypotension, syncope); (2) an association of at least two or more among cutaneous, respiratory, GI, and cardiovascular manifestations associated with exposure to a likely allergen for that patient; or (3) isolated reduced blood pressure after exposure to known allergen for that patient [17]. Food is one of the most common causes of anaphylaxis, with most surveys indicating that food-induced reactions account for 30–50% of anaphylaxis cases in North America, Europe, Asia, and Australia, and for up to 81% of anaphylaxis cases in children [18]. Peanut, tree nuts (walnut, almond, pecan, cashew, hazel nut, Brazil nut, etc.), milk, egg, sesame seeds, fish and shellfish, wheat, and soy are the most common food triggers worldwide; however, any food can potentially trigger anaphylaxis. It is important to underscore that in some severely allergic patients, even a very small amount of food can cause a life-threatening reaction: some individuals can develop symptoms upon exposure to fumes from triggering foods being cooked (e.g., fish) or coming in contact with biological fluids (saliva, seminal fluid) of people who has eaten the food they are allergic to [19,20].

Prevalence of food allergy varies widely in different geographical locations, depending upon the influence of cultures on dietary habits. For example, peanut allergy is one of the most common causes of food anaphylaxis in the United States, the United Kingdom, and Australia, but it is rare in Italy and Spain, where consumption of peanut is significantly lower; conversely, consumption of peach and peach allergy are very frequent in Southern Europe and very rare in Northern Europe [3,21]. Along this line, anaphylactic reactions have been reported after the ingestion of insects such as beetles (mealworm, superworm, silkworm) or carmine red (E120), a color additive used in the food industry, obtained from cochineal scale insects such as *Dactylopius coccus* [22,23,24,25]. Although insects, as well as other foods (e.g., jellyfish), are consumed mainly in selected geographical areas in Eastern Asia, Africa, and Latin America, allergic reactions to these foods might become more common worldwide concomitant with the globalization of dietary habits. In any instance, robust observational findings, coming from consolidated diagnostic procedures, such as properly controlled in vivo challenges (see below), are very difficult to obtain on a large scale. Therefore, inconsistent definitions and methodologies are used in different studies, most of which are based on self-reporting, which generally overestimates food allergy prevalence [3]. A systematic review that included 42 studies conducted in Europe between 2000 and 2012 found a very poor correlation between prevalence estimates in studies relying on self-reported vs. challenge-confirmed food allergies [26].

The foods most consistently associated with self-reported or in vivo challenge-confirmed allergic manifestations (ranging from OAS to systemic anaphylaxis) in the United States and the European Union are listed in Table 2.

Asero and colleagues recently reported on a series of 1110 adolescent and adult Italian patients (mean age 31 years, range 12–79 years) diagnosed with food allergy based on the history of reaction in the presence of positive skin prick test (SPT) or elevated food-specific serum IgE [27]. Anaphylaxis was reported by 5% of food-allergic individuals, with the most common cause being lipid transfer protein (LTP). LTP is a widely cross-reacting plant panallergen. The offending food for LTP-allergic patients was most often peach, but other foods can also include other members of the Rosaceae family of fruits (apple, pear, cherry, plum, apricot, medlar, almond, and strawberry), tree nuts, corn, rice, beer, tomato, spelt, pineapple, and grape [28,29,30]. Importantly, some foods can trigger anaphylaxis with delayed onset after allergen exposure, as in the case of galactose-α-1,3-galactose (α-gal), in which reactions can occur up to 4 to 6–12 h after allergen ingestion [31].

Food-dependent exercise-induced anaphylaxis (FDEIA) is a distinct condition with IgE-mediated mechanisms (such as OAS) that occurs only when the sensitizing food is eaten during the 4 h preceding a physical activity (running, dancing, long walks) or the following hour. The symptoms range from urticaria, angioedema, respiratory, and GI signs to anaphylactic shock [32]. Many different types of foods have been shown to cause FDEIA, including wheat, shellfish, nuts, tomatoes, peanuts, fish, pork, beef, mushrooms, hazelnuts, eggs, peaches, apples, milk, and alcohol, and there are also reports in which the ingestion of two foods together along with exercise are required to trigger a reaction [33]. Reported non-food combination triggers include medications such as non-steroidal anti-inflammatory drugs (NSAID), cold or warm temperatures, menstrual cycle, pollens, and ingestion of dust mites [34]. The pathophysiological mechanisms that lead to a temporary loss of immune tolerance in FDEIA have not been fully established (see [8] for more details).

#### 2.1.1. Diagnostic and Therapeutic Management of IgE-Mediated Food Allergy

An accurate clinical and nutritional anamnesis—that is, a detailed medical history documenting the timing and clinical features of the reactions attributed to food—is the mainstay of the diagnostic process of IgE-mediated food allergy, for which it has a positive predictive value close to 100% [35]. A key diagnostic “dogma” in this setting is that results from any in vitro and in vivo tests have no relevance without relatable clinical manifestations. In children, the initial clinical evaluation should include a thorough examination of the growth status and the level of nutrition, as well as research for associated atopic conditions such as atopic dermatitis (AD), allergic rhinitis, or asthma. Relevant for diagnostic purposes are (1) the anamnestic characteristics of the potential food culprit, i.e., type, quantity, raw vs. cooked, previous tolerance; (2) the circumstances potentially favoring the clinical manifestations (exercise, NSAID or alcohol ingestion, viral illnesses); and (3) the host-specific features, such as a history of atopic diseases and/or presence of co-morbidities. Other important issues to investigate are the response to treatment of the allergic reaction and the time passed since the last episode occurred. In vivo diagnostic tools for immediate hypersensitivity include skin prick test (SPT), prick-by-prick (PBP), elimination diet, and oral provocation test, while in vitro diagnostic tools are based on the determination of specific IgE (sIgE) against food proteins (Table 3).

As for the sensitivity and specificity calculated for SPT and for sIgE tests, it should be considered that the results are expressed as “yes/no”, hence not always directly correlated with clinical outcomes. Sensitivity is typically better than specificity, and, in general, increasing the SPT response size or sIgE level correlates with increasing the likelihood of an allergy. Importantly, diagnosis is not based on a single test. Regarding sIgE, the approach of molecular diagnostics called component-resolved diagnosis (CRD) allows us to identify sIgE for specific proteins, or components, in each food. This allows for an individual risk stratification, avoiding unnecessary nutritional and social restrictions. For example, a finding of sIgE vs. LTP exposes individuals to a risk of moderate to severe reactions as this protein is particularly resistant to peptic digestion and to cooking heat, as opposed to the sensitization to a profilin that generally only causes an OAS.

Another third-level in vitro test is the basophil activation test (BAT), a functional assay that measures the ability of IgE to induce the activation of basophils following allergen binding.

The BAT has the potential to closely replicate in vitro type-I hypersensitivity reactions, mimicking the in vivo responses in allergic individuals exposed to the allergen, and thus it can have clinical applications in the diagnosis and control of allergic disease, alongside research applications. The BAT uses flow cytometry to measure the expression of activation markers induced on the surface of basophils following the cross-linking of IgE bound to the high-affinity IgE receptor (FcεRI) by allergen or anti-IgE antibody. The BAT can be performed using whole blood or isolated leukocytes. The BAT has proven more accurate than sIgE determinations in discriminating between clinically allergic patients and tolerant, sensitized subjects, with specificities ranging between 75 and 100% and sensitivities ranging between 77 and 98% in different studies [36,37,38]. Moreover, the BAT can faithfully predict the severity of allergic reactions, in that patients with more severe reactions show a greater proportion of activated basophils and patients reacting to trace amounts of the allergen show a greater basophil threshold sensitivity (a parameter also referred to as CD_SENS_), i.e., lower concentrations of allergen are sufficient to induce half-maximal expression of basophil activation markers [39,40,41].

The execution of the in vivo tests must be guided by the clinical history reported by the patient and subsequently refined by investigating the sensitization towards the single molecules through the CRD or BAT. An elimination diet for diagnostic purposes is the first in vivo diagnostic step and consists of avoidance of one or more foods suspected of triggering reactions, chosen according to clinical and dietary history coupled with results from appropriate allergy tests such as SPT and serum sIgE levels. The diet must be followed for a set time period, at least until a significant symptom pattern (recurrence or relief) is appreciated: this might be, on average, 2–4 weeks for classical IgE-mediated symptoms, while longer periods, up to 6 weeks, would be required for investigating non-IgE-mediated presentations (see below), as in eosinophilic esophagitis (EoE). The diet must be carefully monitored, and the results obtained must be evaluated to establish or refute the diagnosis, to avoid unnecessary dietary restrictions. If the elimination of an allergen from the diet has a limited or unclear effect, the diet should be carefully re-evaluated to test whether alternative potential food allergens were neglected [35]. Lastly, for most food allergy cases, the oral provocation test is required to confirm the diagnosis, in particular as a double-blind, placebo-controlled food challenge (DBPCFC) protocol, which represents the food allergy diagnostic gold standard [42]. Importantly, oral challenge tests are also essential to monitor the disease course: by demonstrating persistence of reactivity or the acquisition of tolerance, this procedure becomes a major determinant of appropriate dietary indications, ensuring proper nutrition through gradual liberalization in case of newly established tolerance, or an indication of alternative sources of nutrients when strict avoidance is confirmed as necessary.

The clinical management of all forms of food allergy includes both short-term interventions for acute reactions and long-term strategies to minimize the risk of further reactions. Prompt treatment with epinephrine is a cornerstone of therapy of acute severe IgE-mediated food reactions [43]. The patient—or their parents if of pediatric age—should be educated to use epinephrine auto-injectors and other self-medication methods such as antihistamines and steroids for milder reactions. As long-term therapy, the removal of offending foods from the diet (avoidance diet), as well as the ready availability of emergency medication such as epinephrine, are currently the treatment mainstay. However, long-term treatments to prevent reactions to accidental exposure to the food and/or allow its reintroduction in the diet are becoming available. Allergen immunotherapy is an intervention by which an allergic individual is exposed to initially small, gradually increasing quantities of the specific allergen responsible for clinical presentations. The goal is to achieve long-term tolerance or at least sustained unresponsiveness of the immune system, thereby ablating or decreasing, respectively, the probability of an allergic reaction upon allergen re-exposure. In food allergy, major advances have been gained by studies on the efficacy of immunotherapy (IT) approaches, including oral (OIT), sublingual (SLIT), and subcutaneous (SCIT) and epicutaneous immunotherapy (EPIT) [43,44]. Of these, SCIT, which demonstrated a good potential for desensitization in patients with peanut allergy, has been fundamentally abandoned out of concerns for its side effects and overall safety [45,46].

Most data on OIT safety and efficacy come from clinical trials for peanut allergy, the largest of which is a recently published Phase 3 international trial [47,48]. This study showed that six months after achieving a maintenance dose of 300 mg (approximately one peanut), 67.2% of participants receiving active treatment were able to ingest 600 mg or more of peanut protein without dose-limiting symptoms compared to 4% of placebo-treated participants. This and other studies show that under close medical supervision, peanut OIT can be safe and effective for raising the threshold of allergen dose needed to trigger an allergic reaction for many patients. Very recently, the U.S. Food and Drug Administration approved the oral agent, Peanut Allergen Powder^®^, for the treatment of peanut allergy in children at least 4 years old. Other approaches to treating peanut allergy include EPIT: results from a Phase 3 international trial were recently published [49]. This approach of daily allergen exposure through the skin via placement of an adhesive patch embedded with allergen resulted in raising the threshold of allergen dose needed to trigger an allergic reaction for some patients. Side effects typically are limited to local patch site reactions. GI symptoms and anaphylaxis are rare [50].

#### 2.1.2. Nutritional Concerns in IgE-Mediated Food Allergy

A prolonged elimination diet, especially when involving major food groups, must be carefully monitored over time as it can lead to impaired nutrition and decreased quality of life. Ideally, these patients should receive adequate support from a dietician with specific expertise in food allergy, especially when managing infants and children with multiple sensitizations, as tolerance can be different for each food and can change over time. Therefore, periodic reassessments are required to test the development of tolerance and thus resolve to liberalize foods. The management of the exclusion diet must be based on the replacement of foods to which one is allergic with the integration of proteins, vitamins, and minerals to prevent deficiency and taking into account medium and long-term sustainability. By comparison, this management can be complex for children and is more straightforward for adults. Although most IgE-mediated allergies resolve between the age of 5 to 10 years [51,52], they represent a major problem for the health and social life of the pediatric population. Epidemiologically, IgE-mediated allergies are more frequent and long-lasting than other forms of immunologic reactions to food, which might resolve on average within the first 3 years of life. It should also be considered that childhood and adolescence are critical periods from a nutritional standpoint, as a growing individual needs quantities and proportions of macro- and micronutrients that vary greatly during the various stages of development.

Poor substitution of basic foods such as milk, eggs, and wheat can result in increased risk of specific macronutrient deficiencies and insufficient intake for all energy needs. Protein deficient diets can also cause poor growth and related morbidities: to the extreme spectrum, Kwashiorkor has been reported in children on allergen elimination diets [53,54]. In children, weight is an indicator for assessing energy and protein intake with respect to health. However, due to protein deficiency there may be overall reduced growth. Cow’s milk is one of the main foods of the pediatric age (indeed the only one in the first months of life in the infant who is not breastfed), but at the same time it is the main allergen common to all forms of food allergies. In case of cow’s milk allergy (CMA), the first choice in the first years of life is replacement with extensively or partially hydrolyzed cow’s milk formulas that a number of studies demonstrate to be nutritionally adequate and well tolerated [55,56,57,58]. If symptoms persist (anaphylaxis, severe GI bleeding, etc.), an amino acid-based formula may be required. Some of these products contain probiotics supplements (such as *Lactobacillus rhamnosus* GG), which have been shown to reduce symptoms and promote long-term tolerance induction in infants with CMA or other allergies [59].

As the child grows, the use of cow’s milk is sometimes replaced with other animal or vegetable milk source, such as soy-based formula milk. These changes (sometimes prompted by a general “fear of allergies”) can produce harmful nutritional consequences such as calcium and vitamin D deficiencies, or exposure to phytoestrogens and allergic sensitization to soy products [60]. Klemola et al. in a randomized trial found that soy may be less well tolerated than extensively hydrolyzed whey formula, especially among infants younger than 6 months [60]. Two randomized controlled trials suggested that rice hydrolysate formula was well tolerated among infants with CMA and may even reduce the duration of allergy [61,62]. A calcium deficiency is common in children with CMA and must be satisfied with adequate replacement. In 2010, the WAO published “Diagnosis and Rationale for Action Against Cow’s Milk Allergy (DRACMA)”, a set of guidelines that included recommendations for feeding infants and young children with CMA [63]. There are also frequent reports of children developing vitamin D deficiency rickets following dietary restriction [64]. Besides the nutritional needs, it is necessary to consider that adherence to an elimination diet provokes significant stress on young patients and their families, and this leads to restrictions for children and adolescents on attending the school cafeteria, taking school trips, or going at friends’ houses. It must be emphasized that for IgE-mediated food allergies, the elimination diet must be strict, as even small traces of allergen can cause life-threatening reactions. Besides milk, many other food allergy-triggering foods, such as eggs and tree nuts, can be hidden in numerous processed foods that can be easily eaten by an exchange of snacks not carefully evaluated for allergen content by reading the package label. Wheat is the frequent cause of FDEIA in children, in particular in teenage males [18], as adolescents tend to be more physically active through sports or gym activities and rely substantially on wheat and grains for nutrition. In adults, the most common allergens are seafood, peanuts, and tree nuts. Tree nuts include pistachio, pecans, Brazil nuts, cashew, hazelnuts, and walnuts, and people allergic to them often react to more than one variety due to extensive cross-reactivities. Tree nuts have a high content of minerals, proteins, and unsaturated fats, and they carry known beneficial properties for health, such as the cholesterol-lowering properties of walnuts. Although the impact of a necessary avoidance on caloric and nutritional needs can be negligible, the risk of accidental ingestion is substantial as tree nuts are often part of multi-ingredient dishes and present in small quantities or even as a contaminant in many packaged foods; therefore, they are frequently responsible for unexpected reactions due to unintentional ingestion.

A particular group of patients, mostly adults, present reactivity to multiple plant foods through sensitization to LTP, which are plant panallergens whose relevance is mostly limited to the Mediterranean Basin [65,66]. Managing this condition might involve broad nutritional restrictions that are difficult to achieve, in turn leading to severe nutritional deficiency. Often there is only a subclinical sensitization, yet patients must be alerted to the possibility of allergic reactions in the presence of co-factors such as concomitant assumption of alcohol or NSAID or subsequent physical activity.

Lastly, nutritional harms can be caused by erroneous diagnostic procedures leading to inappropriate dietary limitations. In recent years, tests for the diagnosis of food allergy and intolerance not scientifically validated—or even of proven insufficient sensibility and specificity—have gained attention as alternative approaches to identify the causes of signs and symptoms suggestive of food-related manifestations. The use of these tests exposes the patient not only to a possible diagnostic delay of other pathologies but also to serious nutritional imbalances due to the inappropriate elimination of food. This is especially dangerous in children and adolescents where the exclusion of certain foods can lead to growth impairment or serious deficiencies in micronutrients, such as calcium and vitamin D.

### 2.2. Mixed IgE and Non-IgE-Mediated Food Allergy

Eosinophilic GI diseases (EoGD) share a complex pathogenesis triggered by foods in which an IgE-mediated component is integrated with T cell-mediated immunological mechanisms, characterized by a predominant eosinophilic infiltration of the GI tract. It is a rare, heterogeneous, and poorly defined clinical condition that can involve any segment of the GI tract. Patients with EoGD have variable clinical presentations depending on the affected site and the degree of eosinophilic inflammation. These include EoE, eosinophilic gastritis, eosinophilic gastroenteritis, and eosinophilic colitis.

EoE is the most frequent EoGD. EoE is increasingly seen during infancy through adolescence, although it is increasingly being diagnosed in adult age. Failure to thrive is commonly observed in affected children. In older patients, this condition presents clinically with symptoms related to esophageal dysfunction, such as dysphagia, and symptoms mimicking chronic gastroesophageal reflux disease (GERD). Genetic linkage studies, animal models, and the frequency of comorbid allergic disorders link the pathogenesis of EoE with atopy [67]. An elimination diet is a common primary approach, particularly in children, and it might be a treatment option in motivated adults [68,69]. In some cases, EoE appears to be triggered not only by food but also by aeroallergens, while in some instances no clear trigger can be identified [70]. Besides the possible involvement of IgE-dependent immunity, recent studies have also highlighted the presence of IgG4 deposits in the EoE mucosa, hinting at the possible role of this Ig class in the inflammatory response in this condition [71].

AD is a chronically relapsing inflammatory skin disease affecting children and adults and can be present in patients presenting with mixed IgE/cell-mediated food allergies. AD pathogenesis recognizes a complex interaction between skin barrier dysfunction and environmental factors such as irritants, microbes, and allergens. Its clinical presentation and severity vary widely, and diagnosis is not always straightforward, especially in adults. Papules and papulo-vesicles may form large plaques that ooze and crust. They typically affect the face, hands, and extensors, but the scalp, neck, and trunk may also be involved [72].

In some sensitized patients, particularly infants and young children, food allergens can induce urticarial lesions, itching, and eczematous flares, all of which may aggravate AD. The role of food allergens in the pathogenesis and the severity of this condition remains controversial; it is thought to be involved in 30–40% of children with moderate to severe eczema, which generally self-resolves with growth [73].

#### 2.2.1. Diagnostic and Therapeutic Management of Mixed IgE/Non-IgE-Mediated Food Allergy

Clinical presentations and anamnesis for of EoGD may support a first round of IgE-related diagnostics such as SPT and/or sIgE titers, but often the tests are negative or there is poor clinical correlation; hence, an elimination diet followed by an oral food challenge may be needed to identify the causative food allergen(s) [74,75,76]. Currently, diagnosis is based on endoscopic evaluation and bioptic identification of eosinophilic infiltrate [70], the main pathological feature within the involved GI segment. Only 50% of patients show peripheral eosinophilia [75,77,78,79]. AD is only diagnosed on the basis of clinical presentation and history [72]. SPT and sIgE can often identify sensitization to inhaled or food allergens that generally confirm an atopic condition.

In EoGD—where negative or poorly correlated food allergen-related IgE profile are frequently found—an empirical six-food elimination diet is implemented, removing dairy, wheat, egg, soy, nuts, and seafood. In case of no improvement, an amino acid-based elemental diet is adopted. The dietary approach shows good results in upper EoGD, i.e., EoE and eosinophilic gastritis, while it is not as effective in eosinophilic gastroenteritis ad colitis. Once disease remission has been obtained by dietary modification, food groups are slowly reintroduced (at about 3-week intervals for each food group), and endoscopy is performed (approximately every 3 months) to identify sustained remission or disease flare-ups and to document the decreased or absence of mucosal eosinophil infiltration. Another effective therapeutic strategy in EoGD is the use of glucocorticoids, either through specific mucoadherent topical formulation or by swallowing anti-asthmatic inhaled corticosteroids (budesonide, fluticasone, ciclesonide) [80,81,82]. Proton pump inhibitors (PPI) may improve the course of disease in people affected by EoE and eosinophilic gastritis, even when typical gastritis and GERD symptoms are absent [70,81,82]. PPIs used at high dose and continuously can induce remission in about 20–60% of patients affected by EoE with a mechanism that combines their anti-acid and anti-inflammatory activities. Finally, many trials are in progress to evaluate the efficacy of biological drugs in EoGD, one of the best promising being dupilumab, a monoclonal antibody against the interleukin (IL)-4 receptor that inhibits IL-4 and IL-13 signaling, which plays a pivotal role in type 2-driven inflammatory diseases [83].

In AD, the first-line dietary therapy includes a trial of targeted food elimination if sensitization to food is identified by SPT and/or sIgE measurements. However, the correlation with food allergies, while strongest in the first months or years of life, is still relatively low (generally appreciated in no more than 40% of children) [84,85]. Another useful dietary approach in AD seems to be the low-histamine diet, which would help alleviate itching and flare-ups [86]. Besides diet, the therapeutic management of AD is very complex as it includes careful skin care, topical or systemic steroids or immunosuppressants, up to biological drugs (e.g., dupilumab).

#### 2.2.2. Nutritional Concerns in Mixed IgE and Non-IgE-Mediated Food Allergy

Long-term dietary restrictions are the mainstay of treatment in this group of conditions, in particular in patients affected by EoE. The six-food diet involves the avoidance—among others—of foods central in the Western diet as milk and wheat. Wheat in particular is an important source of energy, which should provide between 45 and 65% of the daily energy intake in children. Therefore, in these patients it is essential to provide alternative grains to meet this macronutrient need. Cereals also provide micronutrients (thiamin, niacin, riboflavin, iron, and folic acid) that are not found in fruits and vegetables [87]. Eggs offer excellent quality proteins (they are rich in essential amino acids), vitamins (for example vitamin D) and essential minerals. Legumes provide proteins of good biological quality, although sometimes lacking some essential amino acids. They also contain complex carbohydrates and unsaturated fats, and they are rich in dietary fiber, as well as minerals (such as phosphorus and calcium) and B vitamins. When excluding all these nutrients from the diet, even if temporarily, a proper dietary regimen providing the necessary nutrients from alternative sources is challenging, and patients may clearly benefit from the support of professional nutritionists.

The advantage of dietary approach is to provide effective non-pharmacologic treatment as a long-term disease control option. On the other hand, prolonged food restriction—and, to a greater extent, elemental diets—can pose a risk of nutritional deficiency, can be difficult to manage for patients and families (particularly if nasogastric feeding is required), and can lead to psychological problems and to unnecessary food aversion [88,89]. A relapse upon discontinuation of the diet is common, and long-term EoE recurrence rates while on the same diet remain to be clearly elucidated. This point is salient since adherence to the diet is difficult and can impact the quality of life [90,91]. Another significant concern is due to the lack of non-invasive tests for diagnosis and follow-up of these conditions, which forces affected patients to undergo repeated endoscopies for monitoring the response to therapy.

A targeted food elimination diet guided by SPT or sIgE can improve the course of AD in children. In these cases, cow’s milk and eggs are more frequently avoided, with the implications mentioned above. Soy formulas can be used for the replacement of cow’s milk but do not protect against eczema development. A low-histamine diet is also advised, whose associated nutritional concerns are discussed in Section 3.1.

### 2.3. Non-IgE-Mediated Food Allergy

This group includes celiac disease (CD), food protein-induced enterocolitis syndrome (FPIES), food protein-induced enteropathy (FPE), and food protein-induced allergic proctocolitis (FPIAP) [1,35]. CD is an immune-mediated disorder triggered by dietary gluten, a protein found in cereals such as wheat, rye, and barley. CD is strongly dependent on the genetic background. The disorder is characterized by a small intestinal enteropathy leading to GI as well as extra-GI manifestations and the production of auto-antibodies besides anti-gliadin antibodies, such as anti-endomysium and anti-tissue transglutaminase (TTG) antibodies [92]. The measurement of serum anti-TTG antibodies is very useful for the diagnosis and follow-up of these patients, since they disappear in patients following a gluten-free diet. The seroprevalence of CD was recently estimated at 1.4% worldwide, ranging from 1.1 to 1.8% across geographical areas [93]. Besides CD (not further discussed in this review), this group of food protein-induced diseases have characteristically an early onset (within the first year of life) and GI clinical manifestations. Despite the significant, often dramatic clinical manifestations during the acute phases, the prognosis is favorable, with the majority of patients resolving by age 3–5 years. In acute FPIES, repetitive vomiting, lethargy, and paleness appear from 30 to 240 min after taking the triggering food, which is most commonly cow’s milk, soy, cereals, fish, eggs, poultry/meats, fruit, legumes, or other vegetables. Reactions to multiple foods are not uncommon. Diarrhea can also follow 5 to 10 h later, although this is a less common feature (25–50%). Symptoms may be quite severe, with up to 15% patients experiencing hemodynamic instability. Chronic FPIES typically occurs with persistent exposure to cow’s milk or soy-based formula, and it presents with chronic watery diarrhea (occasionally with blood or mucus), intermittent emesis, abdominal distension, and poor weight gain [94,95,96,97]. Although FPIES generally occurs in early infancy, adult-onset FPIES is now also being increasingly recognized, most frequently triggered by seafood [98,99,100,101]. Finally, a rare occurrence of symptomatic fetal and neonatal FPIES has recently been reported, due to intrauterine sensitization [102,103]. In FPE, symptoms develop in infants shortly after the introduction of cow’s milk in the diet, with chronic diarrhea and features of malabsorption such as steatorrhea and failure to thrive. Vomiting is also frequently reported. FPE is usually transient and typically resolves by 1–2 years of age, as in the case of FPIAP, although in the latter case there is an increased risk of functional GI disorders. FPIAP most often occurs in exclusively breastfed infants within the first weeks of life, because of indirect exposure to maternal dietary protein via breastmilk, although direct feeding can also trigger symptoms. These infants present with bloody, loose stools, sometimes with mucus, but the infants generally appear in well-being [104].

#### 2.3.1. Diagnostic and Therapeutic Management of Non-IgE-Mediated Food Protein-Induced Allergy

Diagnosis of food protein-induced diseases relies, for the most part, on the clinical picture with the exception of FPE, in which histological confirmation is usually required when young infants (<9 months) present symptoms of vomiting and intestinal malabsorption [105]. The diagnosis of FPIES and FPIAP relies on the appreciation of a constellation of concordant symptoms coupled with their resolution upon dietary restrictions of the offending food. An oral food challenge should be strongly considered when only a single episode has occurred, or when the causative food remains elusive, preferably with the documented recurrence of symptoms when foods are re-introduced. Two therapeutic strategies can be adopted, depending on the severity of the symptoms and on the quality of triggering foods: a “bottom-up approach”, in which only causal foods are eliminated, and a “top-down approach”, warranted in most severe cases where failure to thrive and dehydration are prominent. This latter approach consists of an initial avoidance of a wide variety of foods, sometimes starting with an elemental diet, followed by the sequential reintroduction of individual foods. Extensively hydrolyzed cow’s milk formula and amino acid-based formula (AAF) may be useful long-term management strategies for infants with IgE- or non-IgE-mediated cow’s milk allergy (but only 10–20% of the latter patients require AAF). Usually 50% of infants with FPIES caused by cow’s milk develop tolerance by 1 year and 90% by 3 years [106]. FPIES caused by solid foods appears to persist for longer [94]. Sometimes FPIES can arise during breastfeeding; in these cases, if a food trigger is identified, the mother has to follow an elimination diet.

#### 2.3.2. Nutritional Concerns in Non-IgE-Mediated Food Protein-Induced Allergy

The three most common foods causing FPIES are milk, soy, and rice. Rare triggers include other cereals and legumes (peanut, green pea, string bean), sweet potato, squash, carrot, egg white, chicken, turkey, fish, and banana [107]. When milk is the trigger food, it is important to supplement calcium and vitamin D. FPIAP is also typically induced by cow’s milk protein, which requires its elimination from the child’s diet or from the mother’s diet when breastfed infants are affected. Spontaneous resolution is acquired within 1–2 years of age. The most common triggers for FPE are cow’s milk, soy, and rarely chicken, rice and fish [1]; this condition rarely persists beyond 3 years of age. Therefore, a strict surveillance for potential nutritional issues is only required for a limited period, and during follow-up visits it is key to address unnecessary restrictions of milk and dairy products that could further compromise health and quality of life, which is above and beyond the psychological price imposed by the prescribed dietary restriction [108,109].

### 2.4. Pathophysiology of Immunologic Adverse Reactions to Food

**Figure 3:**
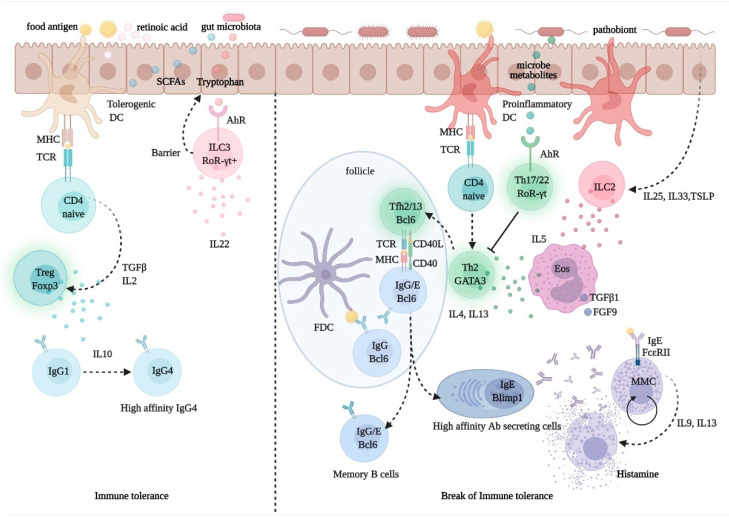
Regulation of immune tolerance in the gut mucosa. Upon processing of dietary fibers, bacterial metabolites, such as short chain fatty acids (SCFA) and retinoic acid (RA), direct the development and function of FoxP3^+^ Treg cells via the interaction with gut epithelial cells and tolerogenic dendritic cells (DCs) with naïve CD4^+^ T cells. The activation and expansion of Treg cells promote the production of the immune regulatory cytokine, IL-10, which foster IgG1 to IgG4 B-cell class switching. Allergen-specific IgG4 B cells produce high-affinity antibodies for food allergens, preventing allergen interactions with mast cell-bound IgE. Microbiota-delivered factors, such as tryptophan-indole catabolites, may directly activate ROR-γt^+^ type-3 innate lymphoid cells (ILC3), via the aryl-hydrocarbon receptor (AhR), and induce the production of IL-22, a cytokine promoting gut epithelial regeneration and barrier integrity. Conversely, upon exposure to pathobionts, DCs and epithelial cells receive danger signals and release cytokines, such as IL-25, IL-33, and thymic stromal lymphopoietin (TSLP); these promote the activation and expansion of ILC2s, which express Th2 cytokines, such as IL4, IL-5, and IL13. While IL-5 promotes eosinophil activation and differentiation and the production of profibrotic factors, such as transforming growth factor (TGF)-β1 and fibroblast growth factor (FGF)-9, IL-13 produced by Th2 cells and T follicular helper (Tfh) 13 cells, is critical for the expression of high-affinity antigen-specific IgE. IgE antibodies interact with FcεRI on mast cells and upon exposure to allergen triggers degranulation and release of histamine, which causes allergy and inflammation.

Despite intensive research efforts over the past several years, the management of allergic diseases and chronic inflammatory conditions, which are on a steady rise in both prevalence and severity, still faces unmet challenges. Central to the pathogenesis of these diseases is the development of a T helper (Th) 2-biased, allergen-specific immune response, characterized by IgE synthesis, eosinophilia, and target organ hyperresponsiveness, and it results from a complex interplay of genetically controlled and environmental factors (Figure 3). The hygiene hypothesis, invoked to explain the disproportionate rise in prevalence of allergic and other inflammatory disorders over the past 40 years, provides a conceptual framework to understanding how a modified environment may pave the way for abnormal, imbalanced immune reactivity in predisposed individuals [110]. However, the factors and pathways mediating predisposition to allergic diseases, collectively referred to as atopy, remain elusive. It is thought that the sign and strength of an immune response to a given antigen (Ag) may reflect an intrinsic, predetermined bias in Ag-specific T cells, yet there is no definitive evidence in support of this theory. In fact, evidence suggests that exposure of non-allergic individuals to allergen may not result in allergen-specific protective, Th1-directed responses, and may instead result in specific tolerance or no response at all [111]. This points to the involvement of an additional or alternative checkpoint(s) in Ag recognition, which may affect immune responses by controlling Ag availability and processing.

Under normal conditions, only minimal amounts of Ag can cross mucosal barriers through the paracellular pathway, a process typically associated with the development of immune tolerance. Ag exposure of inappropriate duration or magnitude may lead to immune-mediated diseases in genetically susceptible subjects. In a few instances, this has been suggested to reflect the intrinsic properties of the antigenic protein. *Dermatophagoides pteronyssinus* (Der p) 1 from house dust mite (HDM), one of the most common indoor aeroallergens, has long been shown to be able to disrupt intercellular tight junctions (TJ) and increase Ag trafficking through bronchial epithelial monolayers [112]. This property, and in general the ability to induce epithelial effector functions, is shared with other allergens—including certain food allergens—and less specific triggers such as detergents and microplastics [113,114,115]. However, allergic sensitization may be facilitated in the presence of intrinsic barrier defects, as evidenced in genetic studies showing the significant association of filaggrin (FLG) loss-of-function mutants, impaired skin barrier function, and the development of allergic disease [116]. Increasing evidence suggests that epicutaneous sensitization may in fact promote the subsequent development of allergic diseases, especially food allergy, in children with AD, thus contributing to the progression of the atopic march [117,118].

As amply documented in a wealth of studies conducted over the past 20 years, the composition and diversity of the microbial communities lining all body surfaces, collectively referred to as microbiota, represent a major, critical variable in the regulation of barrier competence and adaptive and innate responses [119,120]. The gut microbiota is successfully seeded early in life, by colonization from maternal vaginal and breast communities at birth and during lactation [121]. Subsequently, throughout adult life, the microbiota is significantly influenced by dietary habits [122]. Host-microbiota interactions are known to have a critical impact on multiple components of the immune system, contributing to immune homeostasis and susceptibility to infectious and inflammatory diseases. Allergic inflammation resulting from skewed activation of Th2 clones is typically enhanced in germ-free animals, suggesting a major role for gut colonization in the development of balanced Th1/Th2 responses [123]. Likewise, reduced Th1 responses and an increased predisposition to develop allergic disease can be observed in infants delivered by cesarean section, associated with a delayed gut colonization of symbiont species and a less diverse microbial community [124]. In fact, the risk of developing unbalanced immune responses in infancy and childhood, resulting in allergic and autoimmune disease, has also been linked to the maternal diet, particularly during pregnancy and lactation. In support of this theory, recent studies have documented the increased levels of food allergen-specific IgE and IgG antibodies in the offspring of mothers who were prescribed gestational-targeted or exclusion diets [125], and, conversely, an overall reduced risk for immune dysfunction was found following maternal supplementation with probiotics (reviewed in [126]). These overall studies provide factual evidence in support of the hygiene hypothesis, whereby exposure to declining environmental biodiversity, by adversely affecting the human microbiota and its central functions in immune regulation, would primarily account for the rising prevalence of allergic and other chronic inflammatory diseases [127,128].

Highlighted in several studies are the interactions of FoxP3^+^ T regulatory (Treg) cells, a CD4^+^ T-cell subset critically involved in immune homeostasis and tolerance, with microbiota-delivered signals. A sufficiently diverse microbial community may promote the activation and expansion of Treg cells and the production of the immune regulatory cytokine, IL-10, via the interaction of certain bacterial components with Toll-like receptors (TLR) or other pattern recognition receptors (PRR) [129]. Bacterial metabolites, e.g., butyrate and other short chain fatty acids (SCFA) generated upon the processing of dietary fibers, can also direct the development and function of Treg cells via the interaction with gut epithelial cells and dendritic cells and the induction of immunomodulatory mediators such as vitamin A metabolite retinoic acid (RA) [130,131]. As documented in animal models of food allergy, concentrations of butyrate, such as those measured in mature human milk, are sufficient to promote gut barrier integrity and IL-10 production, reduce the allergic response, and enhance the desensitizing effect of allergen immunotherapy (AIT) [132,133]. These findings are invoked to explain the beneficial anti-inflammatory, anti-allergic effects of *Lactobacillus* and *Bifidobacterium* probiotic mixtures and of a high-fiber diet [122,130,132,134].

A connection between diet, gut microbiota composition, and allergic inflammation is postulated in several studies [135,136]. Studies in germ-free mice demonstrated that microbiota-delivered factors can regulate Th2-driven immunity through the induction of Th17 cells and of a subset of Treg cells expressing the Th17 signature factor, retinoid-related orphan receptor (ROR)-γt [137]. *Lactobacillus* strains and other symbiotic species, through the production of tryptophan-indole catabolites, may directly activate these cells, as well as ROR-γt^+^ type 3 innate lymphoid cells (ILC3), via the aryl-hydrocarbon receptor (AHR), and induce the production of IL-22, a cytokine-promoting gut epithelial regeneration, barrier integrity, and the secretion of antimicrobial peptides [134,138]. In addition, IL-1β produced by intestinal macrophages sensing microbial signals can induce the release of granulocyte-macrophage colony-stimulating factor (GM-CSF) from nearby ROR-γt^+^ ILC3, which in turn upregulates RA and IL-10 production from dendritic cells and macrophages, further contributing in the maintenance of tolerance to dietary antigens [139].

The central role of innate immunity in the integration of the environmental signals involved in the development and maintenance of natural tolerance to foods and other antigens has emerged convincingly in recent years. As shown in a cohort study of egg-allergic infants, a distinctive cytokine signature is detected early in life in circulating monocytes and dendritic cells, which is predictive of persistent food allergy in childhood [140]. On the other hand, innate immune profiles in children eventually outgrowing their allergy were directly related to serum levels of vitamin D, further stressing the importance of this nutrient in the development of natural tolerance in childhood [140]. Cytokines released by dendritic cells and epithelial cells upon exposure to pathobiont-delivered danger signals, including IL-25, IL-33, and thymic stromal lymphopoietin (TSLP)—collectively referred to as alarmins—directly contribute to allergic inflammation via the direct activation and expansion of ILC2, an innate lymphoid subset that expresses substantial amounts of Th2 cytokines [141]. The pathophysiologic role of ILC2 in allergic disease has been demonstrated in several models [142,143,144]. These cells, either on their own or in a complex amplifying loop with Th2 cells, may promote and enhance the expression of food-specific IgE in switched and unswitched B cells mainly via the production of IL-4 and IL-13 [143,144,145].

The critical role of IL-13 and related Th2 cytokines in allergic sensitization and its clinical manifestations is emphasized in a recent study documenting that a subset of IL-13-producing T follicular helper (Tfh) cells, termed Tfh13, is critically required for the expression of high-affinity specific IgE and the subsequent development of severe anaphylaxis [146]. Tfh cells, a specialized CD4^+^ T-cell subset defined by expression of the nuclear factor Bcl6 and of the cytokine, IL-21, are key players for the development of switched, memory B cells and plasma cells in germinal centers [147]. While both IL-13 and IL-4 contribute to IgE class-switch recombination and allergic inflammation in part via the interaction with shared receptors, their expression is driven by diverging mechanisms, possibly reflecting their unique involvement in distinct aspects of the allergic response [148,149,150]. Regardless, lineage tracing experiments conclusively demonstrated that in most instances, the switched, allergen-specific IgG^+^ B cells are the precursors of IgE-expressing B cells and IgE antibody-secreting plasma cells [151]. In situ IgE class switching and IgE production have been documented in the respiratory and gastroenteric mucosa in response to such environmental signals, as allergen exposure and microbial superantigens [152,153]. Importantly, single-cell transcriptomic analyses reveal virtually absent IgE^+^ memory B cells in most individuals, whereby most IgE-producing cells are represented by plasma cells [154]. Taken together, these findings suggest that persisting levels of specific IgE in patients with chronic allergies may only be ensured by continuous plasma blast generation via sequential switching from an IgG^+^ memory pool [155,156].

A marked reduction in serum IgE titers was documented in AD patients treated with dupilumab, a monoclonal antibody that blocks IL-4 and IL-13 interaction with their shared receptor [157]. This is consistent with the idea that a significant proportion of IgE are secreted from newly switched, short-lived plasma blasts, and that interfering with Th2 or ILC2 activation and downstream effector signals might hence greatly contribute to restoring tolerance to common allergens. Current AIT protocols, including OIT, are indeed aimed at counteracting these responses via the induction of allergen-specific Treg cells [158]. However, a prospective decrease in specific IgE levels is not a sufficient predictor of clinical outcome in AIT protocols (reviewed in [159]), whereas a rise in other antibody classes, namely IgA, IgG1, and especially IgG4, is more consistently observed [160,161]. Switching to IgG4 is promoted by IL-10, a cytokine produced at higher levels in patients receiving AIT [162]. Such allergen-specific IgG4 undergo increased somatic mutation relative to IgE in these patients, resulting in the expression of antibodies with higher affinity for allergens and is hence more effective at preventing allergen interactions with mast cell-bound IgE [163].

IL-10-producing, immunosuppressive B regulatory cells (Breg) have been recently demonstrated, which were found to be expanded and contribute to peripheral allergen tolerance in patients receiving AIT [164,165]. Interestingly, a subset of Breg cells, termed BR1, were found to be the exclusive source of specific IgG4 and a precursor of IgG4-secreting plasma blasts, in subjects displaying spontaneous or AIT-acquired tolerance to allergen [166]. The recently discovered mutual interactions of effector and regulatory B cells with microbiota components further stress the relative importance of these cells in immune homeostasis in the gut and other mucosal surfaces and the pathophysiology of food allergy and other diseases associated with imbalanced, aberrant responses to environmental antigens.

While most immune-mediated food allergies are associated with predominant Th2-driven, IgE-mediated responses, the development of variably related conditions as eosinophilic esophagitis, FPIES, FPE, FPIAP, and CD, recognizes distinct and relatively complex immune mechanisms. EoE is also mediated by a prevalent, Th2-biased immune response [167]. In particular, elevated levels of IL-5 promote eosinophil differentiation and trafficking to the esophagus [168] and, together with IL-9, are responsible for the progressive eosinophilia and mastocytosis typically observed in the esophageal mucosa in affected patients. Activated eosinophils and mast cells can in turn produce profibrotic factors (such as the transforming growth factor (TGF)-β1 and the fibroblast growth factor (FGF)-9), which cause remodeling of the esophageal epithelium and subepithelium and are responsible for the characteristic symptoms and complications of this condition [169]. However, the association of EoE’s clinical picture with IgE-dominated specific responses to food is not entirely clear. In some cases, EoE symptoms also appear to be triggered by airborne allergens, and in quite a few cases no clear trigger can be identified [70]. Moreover, recent studies also highlighted the presence of IgG4 deposits in the esophageal mucosa, suggesting their possible contribution to the inflammatory response in EoE [71].

In FPIES, a specific T-cell response to causative food antigens leads to TNF-α secretion, which initiates the systemic activation of monocytes, eosinophils, neutrophils, and natural killer cells, resulting in inflammation and increased permeability of the GI mucosa [170,171]. In FPE, the jejunal mucosa is damaged by infiltrating T cells that mostly exhibit a cytotoxic, CD8^+^ effector phenotype and a γδ TCR, causing malabsorption [172]. FPIAP is characterized by a dense eosinophilic infiltration of the rectosigmoid mucosa and typically affects breastfed infants, suggesting the possible role of immunologic components found in breastmilk, such as secretory Igs specific for dietary proteins [173]. Finally, in CD, deamidated α-gliadin-derived peptides are presented by HLA-DQ2/DQ8 complexes of APC in genetically predisposed individuals. Following activation, α-gliadin-specific T cells migrate from the lamina propria into the subepithelial area and begin to produce various pro-inflammatory cytokines, such as IFN-γ and TNF-α. Activated cytotoxic T cells also produce molecules, such as Fas ligand and granzymes, which promote apoptosis of nearby enterocytes. These events combined trigger an extensive immune reaction that causes pathological tissue alterations, resulting in damage of the small intestinal mucosa, villous atrophy, and malabsorption [174]. The ensuing activation of B cells leads to the production of anti-gliadin, anti-endomysium, and anti-TTG antibodies [175]. The presence of anti-TTG antibodies in serum is very useful for diagnosis and for times during the follow-up since they disappear from the serum of patients when they are on a gluten-free diet. However, it is unclear whether they are responsible for the damage to the mucosa or are rather its consequence [174].

## 3. Non-Immunologic Adverse Reactions to Food

Non-allergic food reactions have been also defined as non-allergic food hypersensitivity. In recent years, the term “intolerance” has been often abused to define a wide range of disorders related to the intake of different foods. Multiple and authoritative alerts, both scientific and institutional, ask insistently to review the terminology to place the complex mosaic of these disorders in the more correct clinical definition of “non-immunological adverse reactions to food”. The cornerstone of this renewed need is the “man–food” relationship, now more than ever oriented to combine, with growing interest, nutrition and scientific methodologies of analysis, design, and development of new “personalized” nutritional strategies, with primary respect for the dietary habits and health of each citizen/patient. As already underlined for food allergies, there is a discrepancy between the perceived prevalence of food-related adverse effects, which are extremely common, and the true prevalence of non-immunological food reactions within these events, which is difficult to measure. A recent U.S.-based survey among adult internet users showed that the prevalence of self-reported food intolerance was 24.8% [176]. The very existence and exact prevalence of food intolerances, such as those associated to monosodium glutamate, histamine, as well as non-celiac gluten sensitivity, continue to be highly debated. Along these lines, the real prevalence of lactose intolerance is indeed unknown due to the lack of standardized testing in large, carefully selected populations; conversely, lactose maldigestion affects nearly 65% of the general population [177]. Another gap in our scientific knowledge on “real” intolerances is the absence of validated diagnostic tests, which further impedes active research and correct patient management.

**Figure 4:**
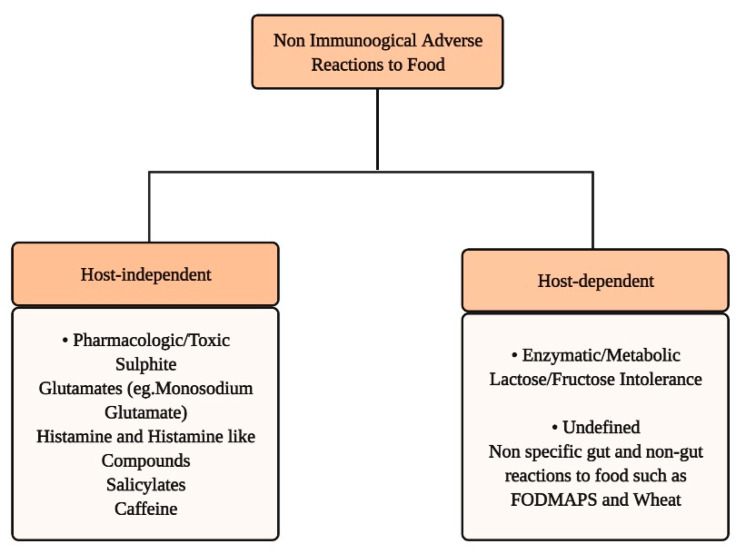
Non-immunologic adverse reactions to food.

The non-immunological adverse reactions to food have been broadly divided between host-independent and host-dependent (Figure 4), due to their heterogeneous pathophysiological mechanisms [178]. The trait d’union is the lack of identifiable underlying immune mechanism. Some food intolerances cannot be readily explained even by currently understood pathophysiological mechanisms and are therefore subclassified as undefined; exemplary for this are the food intolerances reported by irritable bowel syndrome (IBS) patients. In general, the clinical manifestations of food intolerance involve more than one organ or system; however, the spectrum of GI symptoms such as abdominal pain, bloating, abdominal distension, flatulence, and diarrhea is prevalent and very common. In comparison to immune-based food allergies, the amount of ingested triggering food tends to be more directly related to the severity of symptoms. Differently from food allergies, where traces of food allergens may trigger severe reactions, non-allergic food reactions are consistently dose-dependent.

### 3.1. Host-Independent Non-Immunologic Adverse Reactions to Food

Thousands of different chemicals with potential pharmacological activity are present in food, in part added to preserve it and to improve its taste or appearance. They can be natural food chemicals, such as vasoactive amines (e.g., histamine) and salicylates, or food additives, such as glutamates (e.g., monosodium glutamate), sulfites, and benzoates (Figure 4). In some cases, these reactions may mimic reactions typical of an immunologic response. However, unlike true food allergy, there is a delay in symptom onset, a prolonged symptomatic phase, and negative sIgE serology [178]. The most common clinical manifestations of non-immunologic adverse reactions are chronic urticaria or angioedema [179], but a wide spectrum of other clinical features are reported, ranging from atopic eczema, hypotension, flushing, headache, and asthma, along with GI symptoms [180]. Sometimes life-threatening anaphylactoid or severe anaphylactic reactions might occur. The potential pathophysiological mechanisms underlying these non-immunological adverse reactions are under-investigated, and more data are needed to elucidate specific mechanisms of action of sensitivities to specific food additives [181].

Natural food chemicals: Among pharmacologic food intolerances, histamine intolerance or sensitivity to dietary histamine first appeared in the medical literature in the 1980s and is increasingly viewed as a demonstrable disorder [182]. It is believed to arise from an impaired or slowed histamine degradation pathway in the intestine due to low diamine oxidase (DAO) activity, leading to its accumulation in plasma and the appearance of adverse effects, particularly in cases of high dietary intake. This causal link, however, has yet to be proven [183]. Clinical manifestations of histamine intolerance consist of a wide range of non-specific GI and extraintestinal symptoms, due to the ubiquitous distribution of histamine receptors in different organs and tissues of the body. The most frequent and severe manifestations are within the spectrum of GI symptoms, followed by neurological (such as dizziness and headaches) and by cardiovascular signs and symptoms (palpitations, tachycardia, drop in blood pressure); rarer ones are respiratory presentations such as chronic rhinorrhea, sneezing, dyspnea, and dermatological ones with itching, flushing of the face and/or body, urticaria, and angioedema, which is very rare [184]. This pleomorphic presentation contributes to the difficulty in achieving consensus on the diagnostic criteria for histamine intolerance [183]. The lack of a reliant diagnostic system has an impact on the currently reported incidence of histamine intolerance, estimated at around 1–3% of the population. This will possibly increase as more knowledge and diagnostic tools for histamine intolerance become available [185].

Food additives: Significant levels of sulfite, among others, are present in white wine and dried fruits. Sulfites are also added to other foods to enhance crispness or prevent mold growth, while FDA banned sulfites as spray-on preservatives for fresh fruits and vegetables. After their ingestion, adverse reactions have been reported most commonly in susceptible individuals, such as patients with asthma. The proposed mechanisms include inhalation of sulfur dioxide generated from ingested sulfites, deficiency of sulfite oxidase, and IgE-mediated reactions [186]. Additionally, it has been proposed that the parasympathetic system may be involved, whereby inadequate sulfite oxidase results in accumulation of sulfite, causing cholinergic-mediated bronchoconstriction [187]. Another food additive considered a culprit for adverse non-immunological reactions is monosodium glutamate (MSG-E621), which is commonly added as a savory enhancer. However, food high in natural bound or free-form glutamate is metabolized in the same way of MSG, and no studies have demonstrated that it can cause the same symptoms as MSG-added foods [186].

#### 3.1.1. Diagnostic and Therapeutic Management of Host-Independent Reactions to Food

When an adverse reaction to a chemical or natural food additive is suspected, the diagnosis begins with a detailed medical history and a careful collection of the symptoms, including manifestations of atopy. Patterns indicative of intolerance that should be investigated can be multiple: a history of symptoms triggered by several unrelated foods, or to a specific food tolerated when home-cooked but not when commercially prepared, as well as aggravation of a pre-existing disease without a clear cause. The next step is to rule out a “hidden” food trigger. A “food and symptoms diary” can be useful in the diagnostic process, as it helps to rule out a hidden food trigger while introducing the need to check the food labels. There are currently no diagnostic tests available to assess food additive or chemical sensitivity. A double-blinded, placebo-controlled oral challenge (DBPCFC) is considered by some to be the standard criterion for identifying food-related intolerance, despite the inherent challenges that accompany clinical trials of nutrition-based interventions [176]. Before performing the DBPCFC, adhesion to an additive-free diet (no more than 4 weeks) can be considered, to confirm the suspicion of an adverse reaction to food additives if the patient’s symptoms or manifestations improve. The next step is an initial oral challenge trial with multiple additives, which is performed in order to reduce the number of challenges. In case of a positive result with symptoms elicitation, the components of the challenge mixture should be tested separately, in order to identify the food additive responsible of the clinical manifestations. Due to the large number of potential culprits, the re-challenge process can be lengthy, and it is advisable that patients are supervised by a dietitian during the initial restriction as well as through the re-challenge phases. Protocols of oral challenge vary considerably among different studies, and to date, there is not a consensus about the doses that should be used for the challenges [188]. After performing the diagnostic tests, if a food additive is considered responsible of the clinical manifestations, the exclusion of the specific additive from the patient’s diet is the effective treatment.

**Figure 5:**
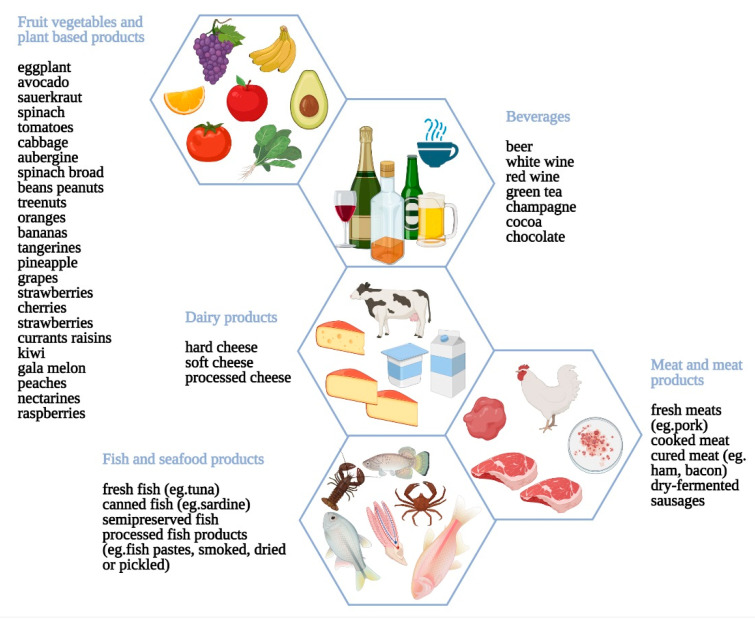
Presence of histamine and other biogenic amines in a wide range of foods.

The combination of the diagnostic criteria currently in use for histamine intolerance includes at least 2 typical clinical manifestations and the diagnostic exclusion of other related disorders: food allergies (by SPT), systemic mastocytosis (by measurement of serum tryptase), concomitant GI diseases as well as the avoidance of DAO-inhibitory drugs. If histamine intolerance is still suspected, then a low-histamine diet excluding foods that, a priori, contain high histamine levels is undertaken [189]. This diet includes a three-stage diet change: in the first 4–6 weeks a strict low-histamine diet to determine symptom response, followed by specific reintroduction of histamine rich-food to determine individual tolerance and eventually a long-term diet personalized to the level of histamine tolerance of each individual. However, as shown in Figure 5, histamine and other biogenic amines are reported to be present in a wide range of foods, and the histamine content in the same food varies significantly depending on maturity, storage time, and processing. Therefore, it is nearly impossible for nutritionists to accurately estimate the amine content to correctly advise patients [190]. A diary of all foods consumed and symptoms experienced is recommended to search for any relationship between the two occurrences. Remission or improvement of symptoms will confirm the diagnosis of histamine intolerance, as well as a possible double-blind placebo controlled oral challenge with increasing histamine dosages to define individual thresholds. A range of complementary tests such as the measurement of DAO activity in the blood or intestinal biopsy or the identification of genetic markers have been investigated; however, evidence-based studies are still lacking. Dietary modifications constitute the therapeutic approach: Food intake is based on a low-histamine diet, although there is no consensus on the list of foods to be excluded. On this basis, a personalized diet should be advisable and should focus primarily on nutrient optimization as well as the interaction with patients to help them differentiate symptoms. If no symptom improvement is observed, all foods should be gradually reintroduced. In some cases, regular use of H1-blockers (antihistamines) is reasonable, although there are no rigorous trials evaluating these agents specifically in patients presumed to have this disorder. Cromolyn, a mast cell stabilizer, has been prescribed in some cases as an oral solution (100–200 mg) taken 20–30 min before meals. Oral supplementation with exogenous DAO from porcine kidney is also used for enhancing the intestinal capacity to degrade dietary histamine [191]. Although few studies have tested the clinical efficacy of this preventive treatment, promising results have been obtained so far. Research is currently also being made to identify new sources of DAO, especially those of plant origin, due to their higher catalytic capacity and other potential productive and commercial advantages.

#### 3.1.2. Nutritional Concerns for Host-Independent Reactions to Food

Presence of these chemicals is widespread in all food groups, and diets avoiding food chemicals are extremely difficult to undertake, as they exclude a wide variety of foods, potentially leading to multiple nutrient deficiencies. Small studies have investigated the effect of single food chemicals. However, these patients had co-morbidities such as IBS or asthma [192,193], and the results cannot be translated to the relatively lower doses found through dietary sources alone. It is also unknown whether the hypersensitivity to one food chemical predisposes an individual to be sensitive to other food chemicals. In this context, it is necessary to keep promoting the multidisciplinary study of these disorders, both from the basic (i.e., analytical chemistry, food science, physiology, and biochemistry) and the clinical perspective, in order to widen the scientific base and the currently available diagnostic and treatment strategies.

### 3.2. Host-Dependent Non-Immunologic Adverse Reactions to Food

Lactose: The disaccharide lactose consists of galactose and glucose, linked by a β-galactoside bond, and it is the culprit of one of the most common enzymatic/metabolic intolerances. In the small intestine, the brush border lactase hydrolyses the disaccharide prior to the absorption of its monosaccharide components. Hereditary forms of lactase deficiency, already apparent at birth, are rare [194]. Conversely, the majority of neonates of every race and ethnicity produce the enzyme to digest lactose in human milk or in standard infant formulas. Nevertheless, lactase synthesis is genetically programmed to decrease after weaning (a condition called lactase non-persistence), resulting in reduced lactase activity in some adults, and an incomplete digestion of lactose [195]. Lactase deficiency can also occur as a consequence of a viral gastroenteritis, inflammatory bowel disease, CD, surgery, or other pathology affecting the small intestine mucosa leading to abnormalities of the brush border, as well as to rapid small intestinal transit or small bowel bacterial overgrowth (secondary or acquired lactase deficiency). Once in the colon, the undigested lactose is digested by colonic bacteria, which form SCFA and produce gasses including hydrogen (H_2_), carbon dioxide (CO_2_), and methane (CH_4_) that have effects on GI function. Lactose intolerance is recognized when individuals with lactose malabsorption report GI symptoms, specifically abdominal pain, bloating, abdominal distension, flatulence, and diarrhea usually starting from 30 min to a few hours after eating or drinking the lactose-containing food [196]. Other symptoms may include GI upset, headaches and migraine, fatigue, musculoskeletal problems, and behavioral changes [197]. These symptoms frequently overlap with symptoms of IBS and fibromyalgia [198]. Although the disease is named lactose “intolerance”, the pathophysiological mechanism, as pointed out, is lactose maldigestion, and the two terms are often interchanged erroneously. The intestinal microbiota plays an important role on how the maldigested lactose is fermented, and this might affect symptoms [197]. Lactase non-persisters may still consume small quantities of dairy foods because lactose works as a prebiotic, inducing the adaptation of colonic microbiome that digests lactose [199]. Conversely, individuals who self-identify as being lactose intolerant may indeed be lactase persisters that wrongly attribute the GI symptoms of (undiagnosed) IBS, or other intestinal disorders [200]. Hypersensitivity to visceral events such as product fermentation is an important consideration [201,202]. Lastly, a history of GI disorders or abdominal surgery play a role in symptoms occurrence.

Fermentable oligo-, di-, mono-saccharides and polyols (FODMAPs) form a group of naturally occurring carbohydrates that present in a wide variety of foods, which have been identified as triggers for symptoms in patients with IBS [203]. The proposed pathophysiological mechanism calls for the osmotic effect of poor absorption of short-chain carbohydrates; this would increase water passage in the intestinal lumen along with an accelerated food passage to the colon, leading to fermentation by colonic bacteria and consequent increased gas production [204,205]. The combined effects of increased water content and gas production in the lumen would cause colonic distention, leading to pain and bloating. The combined effects of increased water content and gas production in the lumen would cause colonic distention, lead-ing to pain and bloating in patients with visceral hypersensitivity, which is considered a hallmark of IBS. Patients with functional GI disorders such as IBS and its subtypes consider bloating as the most bothersome symptoms as well as the least responsive to treatment [206]. It has been suggested that high-FODMAP foods could promote immune activation, aggravating colitis and inducing visceral sensitivity [207]; in support of this hypothesis, a low-FODMAP diet may lead to reductions in urinary histamine and pro-inflammatory cytokines, possibly avoiding mast cell activation [208,209]. However, further data are required to support this evidence.

Gluten is the main storage protein of wheat grains; it results from the combination of a complex of different proteins, mainly gliadin and glutenin. Gluten is the trigger of the immune reaction in CD and wheat allergy, but it has been associated with a wide range of GI symptoms such as bloating, abdominal pain, and bowel habit abnormalities as well as extraintestinal symptoms such as headache, “foggy mind”, fatigue, fibromyalgia, skin rash [210]. This condition has been referred to as “non-celiac gluten sensitivity” (NCGS) [211]. However, although a high number of patients refers to GI symptoms after gluten ingestion, double-blind, placebo-controlled (DBPC) studies demonstrated a very low reproducibility of these symptoms, suggesting that gluten might not be the exclusive culprit [212,213]. In fact, other components of wheat and related cereals may trigger similar clinical manifestations, leading to the adoption of the more comprehensive definition of “non-celiac wheat sensitivity” (NCWS) [212,214,215]. The prevalence of NCWS in the general population is currently based on estimates using various assumptions and ranges from 0.6 to 10.6% [216]. This high variability is mainly due to the lack of specific biomarkers. In fact the mechanisms underlining the NCG/WS are still not fully understood [217] due to the low reproducibility in clinical response, the significant overlap between IBS and gluten-related disorders, the high placebo and nocebo responses; thus, further studies are needed [212,218]. Such studies should include a DBPCC trial with different arms, including pure preparations of gluten, amylase-trypsin inhibitors, and fructans, in parallel with complete wheat preparations and placebo, to fully dissect the impact of the different fractions of wheat in the clinical picture of NCWS [219].

#### 3.2.1. Diagnostic and Therapeutic Management of Host-Independent Reactions to Food

Diagnosis of lactose intolerance can be made with an accurate anamnesis and a clinical evaluation and is confirmed by the resolution of symptoms after avoiding lactose-containing foods for 5 to 7 days. Lactose maldigestion can be detected through multiple diagnostic options such as genetic, enzymatic or breath tests, with the determination of lactase enzyme activity in small bowel biopsies being the most specific [197]. The lactose breath hydrogen test is a non-invasive technique and is the method of choice for testing lactose digestion and patient symptoms. The presence of lactose maldigestion is necessary but not sufficient to diagnose lactose intolerance because the symptom correlation is the key diagnostic element. Management includes dietary lactose restriction opposed to avoidance. Even lactose malabsorbers, who consider themselves to be very lactose intolerant, can actually tolerate moderately large amounts (12–24 g) of lactose (1–2 glasses milk) daily without symptoms [220]. A proposed strategy is to induce a bacterial adaptation or tolerance through changes of intestinal flora using prebiotics or regular consumption of lactose-containing products [199]. It was observed, specifically, that there was an increase in beta-galactosidase activity which enhances digestion and reduces fermentation products [221]. The treatment based on enzyme replacement through the use of exogenous enzymes administered as capsules/tablets before eating has been evaluated by several studies, which confirmed their efficacy [222]. A novel galacto-oligosaccharide, RP-G28, a lactose derivative, was investigated, and half of the study subjects showed complete resolution of abdominal pain at the trial end and at 30 days after treatment completion. Subjects also reported improved lactose tolerance post-treatment with the reintroduction of dairy [221]. When their stool was examined, a relative increase was found in lactose-fermenting bacteria such as *Faecalibacterium, Bifidobacterium,* and *Lactobacillus*. After the reintroduction of dairy, investigators noted a definitive shift in the fecal microbiome to include more Roseburia spp, proving that alterations in diet translate to the gut microbiome [223]. A recent systematic review evaluated the effect of probiotics on lactose intolerance, showing the improvement of some symptoms, such as abdominal cramping, vomiting, bloating, flatulence, and diarrhea [224].

The diet in IBS plays an important therapeutic role, as physicians need to assess eating pattern and diet in IBS patients, and it is well established that IBS patients have a higher perceived food intolerance that makes them avoid foods according to their beliefs, which in turn causes nutritional concerns [225,226]. The United Kingdom National Institute for Health and Care Excellence (NICE) guidelines suggest as first-line treatment in IBS general lifestyle and dietary advice such as the consumption of regular meals and an avoidance of suspected trigger foods and as second line treatment the low-FODMAP diet, with a lactose breath test used to assess the need for lactose restriction [227]. The low-FODMAP diet should be followed by a dietitian and designed as a three-phase diet: a first phase characterized by the short-term (2–8 week) reduction in FODMAP intake, a second phase of re-challenge to assess tolerance, and the last phase of long-term maintenance where only foods that caused symptoms during re-challenge patients were excluded [228,229,230]. Dietary recommendations should be tailored by the dietitian based on the patient’s diet and culture.

#### 3.2.2. Nutritional Concerns for Host-Dependent Reactions to Food

Lactose intolerance may have a relevant impact on nutrition. Dairy foods are valuable sources of protein, calcium, magnesium, potassium, zinc, and vitamin D [231]. Their availability and the relative low cost of dairy products make their consumption more convenient [200]. Despite the evolving knowledge in lactose intolerance, some patients may make unnecessary efforts to avoid all lactose, including lactose used to make up pills. Such misapprehensions about the role of lactose in their symptom production are fueled by an industry promoting largely unnecessary lactase enzyme supplements and alternative milk products. Consumers should be educated by health care providers on the nutritional differences between dairy products and the non-dairy substitutes and should be guided on healthy choices. The food industry can also do its part by improving product labels, indicating lactose content and avoiding misleading claims. Government actions would be wise in introducing legislation that standardizes the definition of “no lactose” and “reduced lactose” and to make lactose content mandatory on nutrition labels.

As with any restrictive diet, a low-FODMAP diet could have potential consequences. High-FODMAP foods usually contain prebiotics; therefore, the reduced intake of FODMAP may result in reductions in overall bacterial abundance, *Bifidobacteria* and *Faecalibacterium prausnitzii* [232,233]. Moreover, the long-term effect of re-challenge on the microbiota is not known. Several studies have raised nutritional concerns about the restrictive phase of the low-FODMAP diet, demonstrating that the reductions in calcium and fibers and long-term effects of this deficiencies are not known [212,232,234,235,236]. In addition, the compliance of patients with the low-FODMAP diet should be further investigated, although the dietitian seems to play a fundamental role [237].

The majority of patients with NCG/WS avoid gluten but also other wheat-related foods that are considered associated to GI symptoms, such as cheese and packaged foods. This dietary regimen tends to include a lower than recommended amount of fiber, carbohydrates, proteins and polyunsaturated fatty acids with possible negative impact on general health [238]. Apart from the nutritional concerns, the economic impact should also be considered, especially where bread and pasta are the foundation of food culture, such as Italy [218]. Gluten-free foods are usually more expensive, and there is no medical reimbursement from public health services.

### 3.3. Psychological Correlates of Food Intolerance

The presence of a food intolerance can impact a patient’s psychological wellbeing. Anxiety, depression, and somatic symptoms are more frequent in patients with food intolerance compared to controls [239,240]. Some studies focusing on psychosocial correlates showed a possible association between food intolerance and younger age, female gender, higher education, and IBS [241]. Furthermore, it was demonstrated that food intolerance could also overlap with food aversions in IBS, and it is possible that this might be significant also in food intolerance [242]. In fact, GI symptoms occur after the ingestion of a food if the intake of a specific food is coincidental with a psychological disturbance, and aversion to that food can be learned. Studies in IBS populations have suggested the need for a better communication and that a positive doctor–patient relationship improves symptom management [243,244]. Thus, it can be hypothesized that this would be successful for patients with food intolerance. Moreover, the presence of a food intolerance associated to GI symptoms may also serve as an argument for food refusal in patients with eating disorders [245,246]. Considering the high prevalence of meal-related symptoms in patients with eating disorders [247], it is crucial that clinicians investigate the presence of a disturbance of eating behavior.

## 4. Conclusions and Future Directions

The public perception of a blurred line between allergy and intolerance carries the costly risk of inappropriate approach of their proper identification and subsequent dietary management, which is the among the key—if not the only in some cases—therapeutic strategy for both types of conditions. This is likely to lead to nutritional gaps in patients with both conditions, as well as to an increase of the burden already carried by the affected individuals, namely the burden of an impaired quality of life and the high economic costs of disease management—beyond the health burden related to their specific disease. In patients suffering from food allergies, the latter is given by the risk of both severe acute anaphylaxis and the worsening of chronic conditions, such as eosinophilic GI diseases and AD. For patients with food intolerance, the health burden is unrelated to anaphylaxis: rather, the difficulties are related to correctly identifying the thousands of chemical or natural food additives or the widespread inclusion of FODMAPs in the Western diet, possibly leading to improper, strict elimination diets that impair the quality of life and lead to nutritional concerns.

When food is the offender, there are several nutritional challenges that patients face in managing the condition causing the problem: delayed, inappropriate, or lack of diagnosis of the nature of the reaction and incorrect dietary management can greatly amplify the impact of the initial problem, leading to nutrition gaps. Initial diagnosis of food allergies requires a correct use of diagnostic procedures, beyond the association of symptoms with sIgE, involving food as a diagnostic tool, that is, time-restricted elimination diets and when necessary, blinded food challenges. These procedures are not carried on in primary care centers, and they are time-consuming and can be expensive; therefore, self-initiated food avoidance trials are often implemented, and such remedy becomes itself an additional source of nutritional deficiencies (summarized in Table 4). An exclusion of the immunologic nature of a food-related reaction based on correct diagnostics should rather be seen as the fastest way to its correct management: importantly, this finding releases the subjects negative to tests from the looming burden of significant risks related to anaphylaxis and liberalizes the consumption of foods that do not carry causative relations with signs and symptoms, thus avoiding potential unnecessary nutritional deficiencies. Accordingly, for patients indeed diagnosed with food allergy, such result allows for a long-term management that sustains a rational dietary approach that includes avoidance only of specific foods and potential desensitization protocols of oral immunotherapy—along with necessary retesting for tolerance and access to life-saving epinephrine injectors.

Several factors currently contribute to an increased awareness—both in the health professional and public communities—of the complex relationship between food intake and the adverse reactions it may cause. An increased understanding of the molecular mechanisms of immunologic and non-immunologic reactions to food, as described in this review and in an ever-growing body of literature, are driving further preclinical and clinical studies on all levels of prevention strategies and on therapeutic interventions. In particular for IgE-mediated food allergies, further developments are aimed at the restoration of a tolerogenic state towards food through intervention on multiple levels: mucosal barrier, microbiota composition, timing of food introduction, up until the development of ground-breaking, allergen-specific T cell-based therapies for severe phenotypes [248].

On a more general level of public health, however, more effort needs to be spent on awareness of the specificity of reactions to food, the importance of appropriate diagnostic procedures, and the necessity of professional support for meeting the nutritional goals when diet needs to be chronically changed due to necessary avoidances. The global epidemic of obesity appears to have somehow overshadowed the nutritional needs and relative care needed for patients suffering from less prevalent food-related diseases such as food allergy and intolerance. The damage brought by this relative lack of professional support on nutritional issues, together with misinformation on causes, diagnostics, and treatments for food-related adverse events has been witnessed by many allergists, gastroenterologists, and nutritionists who have faced patients with food regimens insufficient or even detrimental for health. On a positive note, the increased awareness of the importance of correct nutrition as a key determinant of global health may facilitate a change in the public dissemination of the correct concepts related to the common question: Is it allergy or intolerance?

## Figures and Tables

**Table 1:** Clinical presentations of IgE-mediated food allergy beyond the GI system.

**Cutaneous:** Urticaria-angioedema/pruritus	By food ingestion or contact (an estimated 20% of acute urticarial cases are food allergy-related) [8,9]; severity of IgE-mediated food allergy can be determined by the percentage of the involved skin [10]
**Respiratory:** Rhinoconjunctivitis/asthma	Rarely isolated; commonly associated with other organ/apparatus involvement; can be triggered by allergen ingestion or by inhalation via aerosols (as in baker’s asthma)
**Neurological**	Dizziness or weakness, change in the mental status, unconsciousness (generally associated with anaphylaxis)
**Cardiovascular**	Tachycardia, hypotension, cardiovascular collapse (generally associated with anaphylaxis)

**Table 2:** Prevalence (%) of the eight most common food allergens in adults in the U.S. and the EU [3,21]. Numbers shown represent the average (95% CI) of data collected at centers in different locations.

Food	U.S.	EU	
	Self-Reported	Self-Reported	Challenge-Confirmed
**Cow’s milk**	1.9 (1.8–2.1)	6.0 (5.7–6.4)	0.6 (0.5–0.8)
**Wheat**	0.8 (0.7–0.9)	3.6 (3.0–4.2)	0.2 (0.2–0.3)
**Egg**	0.8 (0.7–0.9)	2.5 (2.3–2.7)	0.1 (0.01–0.2)
**Tree nuts**	1.2 (1.1–1.3)	2.2 (1.8–2.5)	0.3 (0.1–0.4)
**Peanut**	1.8 (1.7–1.9)	1.3 (1.2–1.5)	0.2 (0.2–0.3)
**Fish**	0.9 (0.8–1.0)	1.3 (0.9–1.7)	0.5 (0.08–0.8)
**Shellfish**	2.9 (2.7–3.1)	1.3 (0.9–1.7)	0.1 (0.02–0.2)
**Soy**	0.6 (0.5–0.7)	0.4 (0.3–0.6)	0.1 (0.06–0.3)

**Table 3:** In vivo and in vitro tests for the diagnosis of IgE-mediated food allergies [7].

In Vivo Tests	
**Elimination diet**	This involves an eating plan that omits a food or group of foods believed to cause an adverse reaction. By removing certain foods for a period of time and then reintroducing them during a “challenge” period, it allows the identification of which foods are causing symptoms. The elimination of 6 foods, i.e., eggs, soy, cow’s milk, wheat, seafood, and peanut/tree nuts, can be therapeutic and diagnostic in EoE.
**Oral food challenge (OFC)**	OFC is the gold standard for diagnosis of food allergy. It consists of administering the suspect food at established doses and observing the clinical response in a protected clinical setting.
**Skin prick test (SPT)**	Commercial extracts of allergen are inoculated subcutaneously to detect the presence of sIgE bound to mast cells.
**Skin Prick by Prick (PbP)**	PbP is similar to the SPT but is performed using fresh, cooked or raw food.
**Atopy Patch Test (APT)**	The suspect food is applied directly on the skin using special supports and removed after 48–72 h to study non-IgE (cell-mediated) or mixed IgE/cell-mediated responses.
**In vitro Tests**	
**Total serum IgE (tIgE)**	The total concentration of IgE in the blood is measured; this is useful for assessing the presence of an allergic background but does not identify specific triggers.
**Radioallergoimmunosorbent (RAST) detection of allergen-specific IgE (sIgEs)**	Fluorescent enzyme-labeled antibody assay measures absolute sIgE levels. Values may correlate with the likelihood of clinical reaction for specific foods.
**Component Resolved Diagnosis (CRD)**	CRD is similar to RAST, but it utilizes purified native or recombinant allergens to detect sIgE antibodies against individual allergenic molecules.
**Basophil Activation Test (BAT)**	BAT measures by flow cytometry the expression of activation markers on the surface of basophils following the cross-linking of IgE bound to the high-affinity IgE receptor (FcεRI) by allergen or anti-IgE.

**Table 4:** Nutritional gaps and possible replacement strategies in diets excluding the eight most common IgE-dependent food allergens.

Allergen	Deficiency	Substitute
Cow’s Milk	Calcium, vitamin D, protein, phosphorus, magnesium, potassium, vitamin B12, zinc	Almond milk, oat milk, coconut milk, rice milk, cashew milk, hems milk, macadamia milk
Wheat	Fiber, folate, vitamin B12, selenium, manganese, phosphorus, copper	Rice, quinoa, millet, amaranth, buckwheat, sorghum, teff
Egg	Retinol (vitamin A), riboflavin, thiamin, vitamin B6, vitamin B12, biotin, folate, pantothenic acid, potassium, magnesium, phosphorus, iron, selenium, zinc, iodine	Tofu, mashed banana, yogurt, buttermilk, chia seeds
Tree Nuts	Protein, fat, MUFA, PUFA, linoleic acid, carbohydrates, fiber, calcium, iron, magnesium, phosphorus, potassium, sodium, selenium, zinc, copper, vitamin C, thiamin, riboflavin, niacin, pantothenic acid, vitamin B6, folate, vitamin B12, vitamin A, β-carotene, lycopene, lutein, zeaxanthin, vitamin E	Pumpkin seeds, sunflower seeds, chickpeas, sesame seeds, olives, avocado
Peanut	Protein, fat, fiber, magnesium, folate, vitamin E, copper, arginine	Sunflower seeds, sesame seeds, flax seeds, tree nuts (almonds, cashews, walnuts)
Fish	Omega-fatty acids, proteins, iron, zinc, copper, vitamin B12, vitamin D	Walnuts, flaxseed oil, soy oil, canola oil, egg, sesame butter, leafy green vegetables (spinach, spirulina)
Shellfish	Omega-fatty acids, proteins, irons, zinc, copper, vitamin B12	Coldwater fish (salmon, tuna, mackerel, sardines), egg, nuts, seeds
Soy	Protein, fat, fiber, vitamin C, vitamin K, thiamine, riboflavin, folate, iron, magnesium, phosphorus, potassium, zinc, manganese, copper, vitamin E, niacin, vitamin B6, pantothenic acid	Fresh vegetables, plant proteins, grains
